# Localized radiotherapy of solid tumors using radiopharmaceutical loaded implantable system: insights from a mathematical model

**DOI:** 10.3389/fonc.2024.1320371

**Published:** 2024-02-26

**Authors:** Anahita Piranfar, Mohammad Souri, Arman Rahmim, Madjid Soltani

**Affiliations:** ^1^ Department of Mechanical Engineering, K. N. Toosi University of Technology, Tehran, Iran; ^2^ Department of NanoBiotechnology, Pasteur Institute of Iran, Tehran, Iran; ^3^ Departments of Radiology and Physics, University of British Columbia, Vancouver, BC, Canada; ^4^ Department of Integrative Oncology, BC Cancer Research Institute, Vancouver, BC, Canada; ^5^ Department of Electrical and Computer Engineering, University of Waterloo, Waterloo, ON, Canada; ^6^ Centre for Biotechnology and Bioengineering (CBB), University of Waterloo, Waterloo, ON, Canada; ^7^ Centre for Sustainable Business, International Business University, Toronto, ON, Canada

**Keywords:** implantable drug delivery system, prostate cancer, ^177^ Lu-PSMA, mathematical model, radiopharmaceutical therapy

## Abstract

**Introduction:**

Computational models yield valuable insights into biological interactions not fully elucidated by experimental approaches. This study investigates an innovative spatiotemporal model for simulating the controlled release and dispersion of radiopharmaceutical therapy (RPT) using ^177^Lu-PSMA, a prostate-specific membrane antigen (PSMA) targeted radiopharmaceutical, within solid tumors via a dual-release implantable delivery system. Local delivery of anticancer agents presents a strategic approach to mitigate adverse effects while optimizing therapeutic outcomes.

**Methods:**

This study evaluates various factors impacting RPT efficacy, including hypoxia region extension, binding affinity, and initial drug dosage, employing a novel 3-dimensional computational model. Analysis gauges the influence of these factors on radiopharmaceutical agent concentration within the tumor microenvironment. Furthermore, spatial and temporal radiopharmaceutical distribution within both the tumor and surrounding tissue is explored.

**Results:**

Analysis indicates a significantly higher total concentration area under the curve within the tumor region compared to surrounding normal tissue. Moreover, drug distribution exhibits notably superior efficacy compared to the radiation source. Additionally, low microvascular density in extended hypoxia regions enhances drug availability, facilitating improved binding to PSMA receptors and enhancing therapeutic effectiveness. Reductions in the dissociation constant (K_D_) lead to heightened binding affinity and increased internalized drug concentration. Evaluation of initial radioactivities (7.1×10^7^, 7.1×10^8^, and 7.1×^109^ [Bq]) indicates that an activity of 7.1×10^8^ [Bq] offers a favorable balance between tumor cell elimination and minimal impact on normal tissues.

**Discussion:**

These findings underscore the potential of localized radiopharmaceutical delivery strategies and emphasize the crucial role of released drugs relative to the radiation source (implant) in effective tumor treatment. Decreasing the proximity of the drug to the microvascular network and enhancing its distribution within the tumor promote a more effective therapeutic outcome. The study furnishes valuable insights for future experimental investigations and clinical trials, aiming to refine medication protocols and minimize reliance on *in vivo* testing.

## 1 Introduction

Prostate cancer is a prevalent malignancy globally, ranking second in incidence among all cancers, and responsible for approximately 15% of newly diagnosed tumors in males (1). The primary factor that determines the burden of the disease is its potential for metastasis, which results substantial morbidity and mortality among patients (2). Despite advancements, challenges persist in managing metastatic prostate cancer, prompting a shift to earlier-stage therapies for improved outcomes. Typically, prostate tumors are treated through radiation therapy or surgery intervention, both of which may result in serious side effects and may not consistently yield favorable outcomes (3, 4). Numerous medical interventions are accessible for the management of patients with recurrent and metastatic prostate cancer, including androgen deprivation therapy, chemotherapy, and immune-based treatments (5). As the progression of malignancy ensues, the effectiveness of conventional therapies tends to diminish, resulting in the development of resistance to hormonal manipulations, increased toxicity, or the acquisition of castration resistance (5–7). As such, the development of innovative therapeutic approaches, particularly in early-stage disease, is of utmost importance. The emergence of novel therapeutic agents such as radiopharmaceuticals holds great promise in transforming the management of prostate cancer.

Radiopharmaceutical therapy (RPT) is a type of targeted therapy that employs radiolabeled molecules to selectively target cancer cells while minimizing harm to healthy tissue (8–10). There are several types of RPT, and radioligand therapy is one of them (6, 11). This type of RPT employs a ligand that is labeled with a radioactive substance to specifically target and deliver radiation to cancerous cells. The target receptor on the tumor cell is the sole site where the radiolabeled ligand is intended to interact. The demise of the cancerous cell is attributed to the DNA impairment instigated by the alpha or beta radiation emanating from the radioactive element conjugated to the ligand (12, 13). The utilization of specific ligands results in the preferential accumulation of radiopharmaceutical agents in tumors as compared to normal tissues. Despite the potential harm to adjacent healthy cells, cancer cells are more susceptible to radiation due to their higher rate of proliferation and less efficient DNA repair mechanisms. Promoting RPT through the utilization of ^177^Lutetium-labeled prostate-specific membrane antigen (^177^Lu-PSMA) targeting agents is a prime illustration of radioligand treatment in prostate cancer (11, 14). This method may target prostate cancer cells that express the PSMA protein, producing a more potent therapeutic effect (15). An internalization of ^177^Lu-PSMA is not required since the typical tissue range of ^177^Lu beta-rays is 0.23 mm, much greater than the cell diameter (16). Radiopharmaceuticals have the potential to be administered intravenously, thereby enabling a comprehensive attack on all tumors in cases where prostate cancer has metastasized (17). Administration of the drug intravenously to patients in the early stages of cancer, prior to metastasis, may result in organ toxicity, particularly to the liver and kidneys (18, 19).

As previously stated, radiopharmaceuticals are typically administered intravenously (20). Owing to the constrained allocation of systemic blood flow directed specifically towards the tumor and high clearance rate in circulation, only a small fraction of the total administered dosage successfully reaches the intended tumor site. The remaining dose is distributed throughout the body healthy tissues, potentially leading to unfavorable side-effects and outcomes (21). Numerous medications, including radiopharmaceuticals, exhibit rapid plasma clearance, leading to relatively short half-lives (20, 22) limiting efficacious delivery.

To overcome such shortcomings, novel approaches including use of nanocapsules (23) may aid in reducing these restrictions. Nevertheless, there exist various biological barriers that have the potential to diminish the therapeutic effectiveness of nanoparticles. Organs such as the kidneys, spleen, and liver, along with resident immune cells, participate in innate processes aimed at eliminating foreign substances (23, 24). Consequently, the process of clearance leads to a reduction in the amount of nanoparticles that are delivered to the tumor. Furthermore, the inefficient Enhanced Permeability and Retention (EPR) effect results in a mere 0.6-0.7% accumulation of the injected dose of nanocapsules in the tumor (25, 26). This low level of accumulation significantly hampers the bioavailability required to effectively suppress tumor growth (27, 28). As a result of the challenges faced in the delivery of pharmaceuticals to the tumor site while minimizing damage to healthy tissues, researchers are exploring innovative methods. The potential of biodegradable implants for tumor treatment before metastatic has garnered significant attention (29). The efficacy of localized therapies can be significantly enhanced by elevating the regional concentration of cytotoxic treatments at the intended sites (30). This approach can address the limitations of systemic treatment, such as restricted solubility and transport functionality (29, 30). As such, the utilization of intratumoral biodegradable implants for the delivery of (radio)pharmaceutical therapy to the intended site represents a beneficial strategy for the treatment of solid tumors. The combination of intratumoral biodegradable implants and RPT presents a synergistic approach that holds great potential in revolutionizing the field of oncology.

In the present study, a comprehensive 3D computational model is created for the first time to simulate the dispersion and local release of ^177^Lu-PSMA-617 following the insertion of a dual-release implant into a solid tumor. Understanding the complex bio-drug interactions governing radiopharmaceutical transfer is crucial for deciphering its intricate mechanisms within tissues. Empirically derived models can offer insights into the process, however, achieving a thorough comprehension of the mechanisms governing drug transport and drug-bio interaction over time is a challenging if not insurmountable task. Mathematical and computational models serve as valuable tools for acquiring a comprehensive understanding of the process (31, 32). The utilization of computational models has the potential to reduce the quantity of animal tests, while also decreasing costs and development duration. The utilization of computational models has the potential to forecast the efficacy of drug-loaded implants in solid tumors, thereby enabling researchers and clinicians to enhance implant design and therapeutic response (29, 33).

Current radiopharmaceutical-loaded dual-release implant systems offer several advantages over brachytherapy in the treatment of early-stage prostate tumors. These systems are capable of performing various types of brachytherapy, such as High-dose rate (HDR) implants through burst release, Low-dose rate (LDR) implants through sustained release, and Permanent implants through their biodegradable feature. In contrast to brachytherapy, this technique offers the advantage of biodegradation, thereby eliminating the necessity for removal of the radiation source from the body and providing a less invasive treatment option. Furthermore, the potential for drug leakage from the implant device allows for access to distant areas and the creation of extensive necrotic areas, surpassing the capabilities of brachytherapy. Moreover, the use of radioligands enables internalization by cancer cells, leading to better therapeutic response and irreversible necrosis.

The main goal of this research is to assess the influence of key factors, including hypoxia (as indicated by low microvascular density), on local delivery. Additionally, the study analyzes the impact of the initial amount of ^177^Lu-PSMA and binding affinity on the absorbed dose within the tumor. The present model takes into account specific tumor microenvironment characteristics, encompassing elements like hypoxia and microvascular density, and incorporates the unique properties of ^177^Lu-PSMA. These characteristics play a pivotal role in understanding and optimizing an implantable delivery system for localized radiotherapy. By addressing these parameters, the present study contributes to unraveling the complexities of targeted radiopharmaceutical delivery, aiming for more effective and tailored cancer treatment strategies.

## 2 Methods

**Figure 1:**
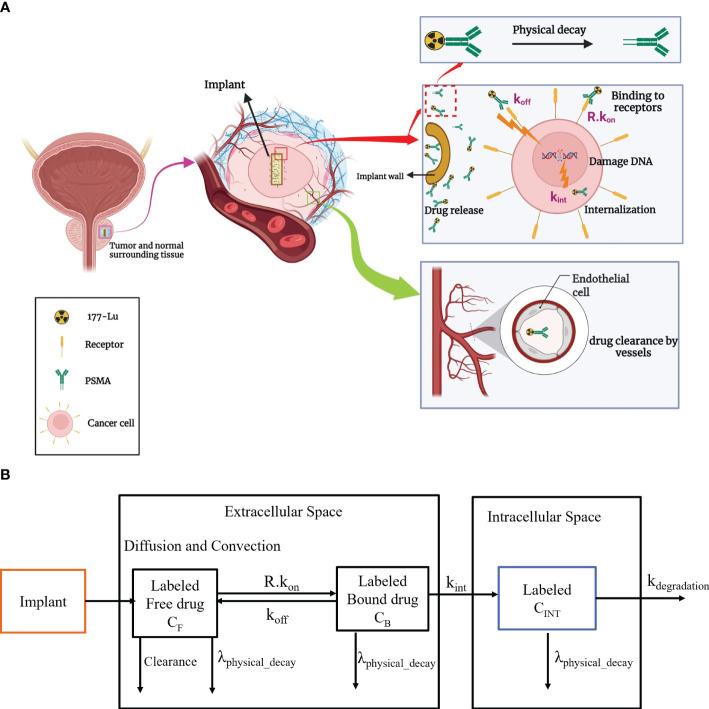
**(A)** An overview of the implantable radiopharmaceutical delivery system for tumor treatment. The therapeutic process involves implant insertion, controlled release of 177Lu-PSMA, dynamic interactions with cell receptors, and subsequent internalization through endocytosis. **(B)** Radiopharmaceutical transport is compartmentalized in implant, interstitial, and intracellular areas, including spatiotemporal modeling. k_on_, Rate of association of drug with receptors; k_off_, Rate of disassociation of drug with receptors; k_int_, Constant of cellular uptake; k_degradation_, rate of degradation of ^177^Lu-PSMA-617.

In this investigation, the radioligand is loaded into an implant that can be inserted in the center of the tumor tissue. In practical, the implant can be located by a catheter (34). The total amount of substance that is loaded into the implant, the diameter and height of the implant, and the release rate of the drug are the key design factors which can be determined based on tumor size. Drug distribution involves ^177^Lu-PSMA release in the interstitium and drug association/disassociation with cell surface receptors at K_ON_ and K_OFF_ rate constants (17). The radionuclide decays by physical decay in all stages. Radiopharmaceuticals in the extracellular space can exit through diffusion into the bloodstream or lymphatic vessels. Yet, dysfunctional lymphatic networks in tumors may hinder this process. Ultimately, radiopharmaceuticals bound to receptors enter cells through endocytosis. This work demonstrates concentration exchange between various biophysical spaces using a multi-compartmental approach. An overview of molecular interaction as a paradigm for radiopharmaceutical administration by implant in the tumor and multi-compartmental model are shown in **Figure 1**. After the release of ^177^Lu-PSMA from the implant, ^177^Lu-PSMA reversibly binds to the receptors present on the surface of cells. Subsequently, ligands may be internalized by the cell and endocytosed.

A comprehensive explanation of equations, parameters, and their corresponding values can be found in the **Supplementary File**. Subsequently, the underlying physics and governing equations are delineated below.

### 2.1 Implant

In this study, drug release is characterized by two phases: an initial burst release, followed by a sustained release. The flux, representing the quantity of ^177^Lu-PSMA released across the implant surface with time, is computed as follows (29) (Equation 1):


(1) $$R_{177lu-PSMA}\left(t\right)=\frac{M_{0}.w_{\infty }}{A}(f.k_{f}e^{-k_{f}t}+\left(1-f\right).k_{s}e^{-k_{s}t})$$

### 2.2 Fluid flow

Tissue interstitial fluid flow is computed employing Darcy’s law (Equation 2), which governs fluid flow through a porous medium, with the inclusion of source and sink terms for biological tissues (Equation 3). The relationship between interstitial fluid pressure (IFP) and interstitial fluid velocity (IFV) within the tissue is established by Darcy’s law equation (35, 36). Additionally, transvascular fluid flow is determined using Starling’s law.


(2) $$v_{i}=-\kappa \nabla P_{i}$$

(3) $$\nabla \cdot v_{i}=\underset{\text{Source term (vessels)}}{\underset{⎵}{\varphi_{B}}}-\underset{\text{Sink term ( lymph vessels)}}{\underset{⎵}{\varphi_{L}}}$$

### 2.3 ^177^Lu-PSMA concentration

The distribution of ^177^Lu-PSMA is elucidated utilizing a multi-compartmental model (**Figure 1B**). A drug that inhibits cancer is released from the implant and diffuses into the surrounding tissue based on convection and diffusion mechanisms (37). Within the interstitial fluid, the transport of radiopharmaceuticals adheres to the Convection-Diffusion-Reaction (CDR) equations (37). Descriptions of the free drug, bound drug, and intracellular drug concentrations are outlined as follows (Equations 4–6):


(4) $$\frac{\partial C_{labeled\_F}}{\partial t}=-\overset{convection}{\overset{⎴}{v\nabla C_{labeled\_F}}}+\overset{diffusion\;}{\overset{⎴}{D_{eff}\nabla^{2}C_{labeled\_F}}}-\frac{(R_{max})k_{on}C_{labeled\_F}}{\psi }+k_{off}C_{B}-FC_{labeled}-\lambda_{\text{lu}177}C_{labeled\_F}$$

(5) $$\frac{\partial C_{B}}{\partial t}=\frac{(R_{max})k_{on}C_{labeled-F}}{\psi }-k_{off}C_{B}-k_{\mathrm{int}}C_{B}-\lambda_{\text{lu}177}C_{\text{B}}$$

(6) $$\frac{\partial C_{\mathrm{int}}}{\partial t}=k_{\mathrm{int}}C_{B}-k_{\text{deg}}C_{\mathrm{int}}-\lambda_{\text{lu}177}C_{\mathrm{int}}$$

### 2.4 Absorbed dose

In radiopharmaceutical therapy, the absorbed dose is the amount of ionizing radiation energy deposited per unit mass of a material (38). To calculate the absorbed for tumor the following equations are used (17, 39, 40) (Equations 7, 8):


(7) $${\overset{•}{D}}_{i}(t)=A_{i}(t)\cdot S_{i\leftarrow i}=A_{0}\cdot a_{i}(t)\cdot S_{i\leftarrow i}$$

(8) $$D_{i}(T)\;=\int_{0}^{T}{\overset{•}{D}}_{i}(t)dt=A_{0}\cdot {\tilde{a}}_{i}(T)\cdot S_{i\leftarrow i}$$

### 2.5 Cell survival probability

Cell survival probability refers to the likelihood that a particular cell will survive following exposure to a specific dose of ionizing radiation (41). To calculate this probability, linear quadratic models are employed, which consider the absorbed dose (42) (Equation 9).


(9) $$P_{S}=e^{-\alpha D-\beta D^{2}}$$

## 3 Results

### 3.1 Validation

**Figure 2:**
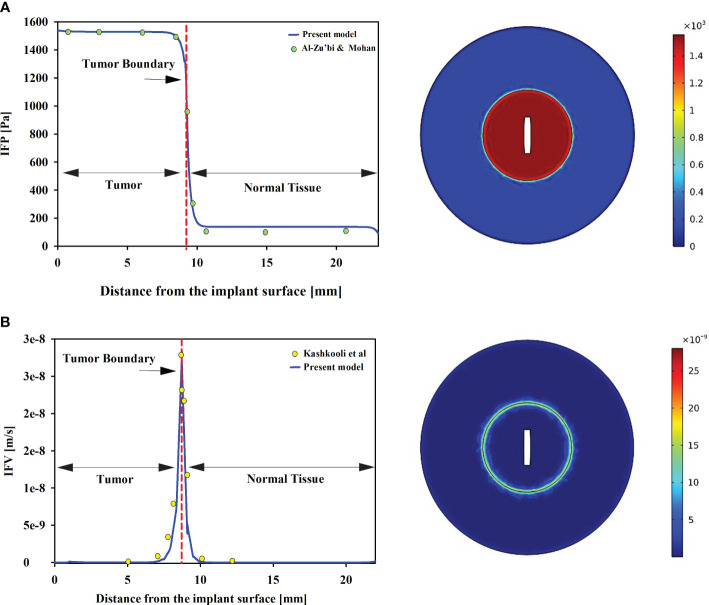
**(A)** IFP in tumor and normal tissue. Tumors exhibit significantly higher IFP compared to normal tissue. Furthermore, uniform distributions of IFP are observed in both tumor and host tissues. Our pressure distribution is validated against Al-Zu’bi and Mohan’s numerical models (29). **(B)** IFV in the tumor and normal tissues. The results show good agreement with Kashkooli et al.’s findings (43). The IFV is also uniform in tumor and normal tissue, with a minimum value, while the maximum value is observed at the tumor and normal tissue border.

To ensure the reliability of the present model, qualitative and quantitative comparisons have been made with findings from experimental studies. As the combination of implant and radioligand therapy is introduced for the first time, there are no available results to validate alterations in concentration. Consequently, other influencing factors are scrutinized to evaluate the outcomes and concentration values. In the context of drug delivery within the tumor microenvironment, the intricate interplay of interstitial fluid flow assumes a paramount role in determining therapeutic efficacy. Key parameters governing this flow are IFP and IFV. In numerical models, Darcy’s law is commonly employed to describe flow in biological tissues, accounting for tissue porosity. The visual representation in **Figure 2** illustrates the spatial distribution of IFP and IFV. The maximum IFP in the tumor and normal tissue is nearly 1500 and 100 Pa. Furthermore, the IFP in normal tissue is considerably lower than that in tumor. Along the tumor-normal tissue border, a pressure gradient is observed within a confined region. The high leakage of blood microvessels and the dysfunction of lymphatic microvessels contribute to an increase in IFP, which becomes approximately equal to the pressure within the microvessels (44). Significantly, the disparity between tissue pressure and microvascular pressure emerges as a pivotal factor influencing the transvascular exchange of therapeutic agents, with direct implications for drug delivery through convection.

To ensure the validation of the model, a comprehensive comparison was conducted between IFP and IFV obtained from the model and those reported in other relevant studies. Experimental investigations have provided data on the mean spatial pressure in tumor and normal tissues, with reported values ranging from 586 to 4200 [Pa] and -400 to 800 [Pa] (45, 46). The present model’s predictions align well with these experimental findings, indicating spatial pressures of 1550 [Pa] in tumor tissue and 65 [Pa] in normal tissue, as depicted in **Figure 2**. These results not only corroborate the experimental studies but also demonstrate consistency with other numerical investigations (29, 43). Zhan et al. employed a 3D model to examine IFP in prostate tumors and the adjacent tissue (47). Their study reported a range of IFP values for tumors between 1400-1500 [Pa], while normal tissue exhibited values around 50 [Pa]. Remarkably, the present findings align closely with the outcomes of Zhan et al., underscoring the consistency and reliability of the present results. Additionally, Zhang’s investigation (48) also supports the present observations, emphasizing that interstitial fluid pressure in prostate tumors tends to be higher than that in the surrounding tissue, a correlation that resonates with the outcomes of the present study. In contrast to IFP, the IFV exhibits its lowest values within both tumor and normal tissues, while the highest magnitudes occur exclusively at the tumor-normal tissue interface. Remarkably, the present model predicts an IFV on the order of 10^-8^ m/s, which is supported by a previous numerical investigation (49, 50), further enhancing the credibility of the present model’s predictions.

### 3.2 Effect of extension of hypoxia region on the distribution of therapeutic agents

**Figure 3:**
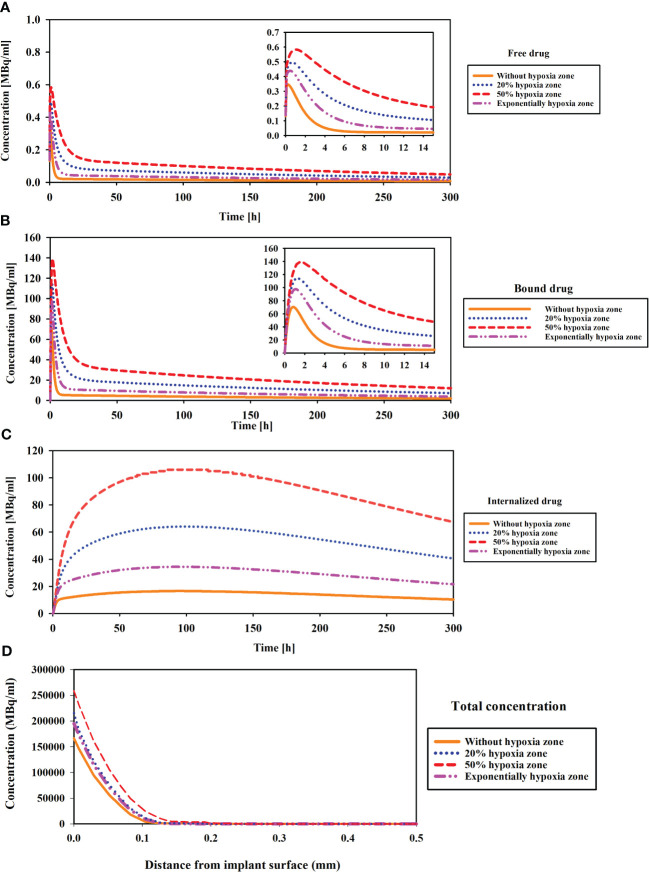
Comparison of concentration changes compared to the hypoxia region in different compartments of CDR model. **(A)** The concentration of released drug from implant (Free drug), **(B)** Binding drug to receptors, **(C)** Internalized drug, and **(D)** The total concentration. Hypoxia with low microvascular density influences ^177^Lu-PSMA-617 concentration: increased accumulation in extracellular space, enhanced binding to cell surface receptors, elevated internalization within tumor cells and tumor penetration.

Hypoxia, referring to low density of microvascular networks within the tumor microenvironment, significantly influences the concentration of ^177^Lu-PSMA-617 in various compartments. The distribution of radiopharmaceutical agents in relation to hypoxic regions is analyzed in **Figure 3**, shedding light on the impact of hypoxia on ^177^Lu-PSMA-617 behavior. As depicted in **Figure 3A**, an increase in tumor hypoxia leads to a rise in the concentration of Free ^177^Lu-PSMA-617. In the extracellular space, hypoxia causes a reduction in intratumoral blood vessels, hindering the removal of free ^177^Lu-PSMA-617 from this region (42). Moreover, as illustrated in **Figure 3A**, it becomes apparent that in the absence of hypoxia, the concentration of free drug in the interstitial space diminishes and undergoes clearance with a more pronounced slope. This is attributed to the heightened “sink” effect, which is responsible for robustly clearing free drugs from the extracellular space, ultimately resulting in a reduced accumulation of ^177^Lu-PSMA-617 in this region.

The results demonstrate the peak concentration are observed in the case of 50% hypoxia (**Figure 3B**). This escalation is attributed to the impact of extension of hypoxic zone and low microvascular density on diminishing the clearance of free ^177^Lu-PSMA-617, leading to its accumulation and heightened binding to PSMA receptors, consequently elevating the concentrations of bound PSMA. As shown in **Figure 3C**, when the hypoxia level reaches 50%, the drug concentration rises with a much steeper slope compared to the scenario without hypoxia, ultimately leading to a higher peak concentration. Furthermore, **Figure 3D** illustrates the total concentration of ^177^Lu-PSMA versus the distance from the implant surface in the tumor (1 h after implant placement). The 50% hypoxic region is characterized by the highest penetration, attributed to lower vascular density. This condition leads to a reduced clearance of radiopharmaceuticals from the tumor, facilitating deeper penetration of the ^177^Lu-PSMA.

**Figure 4:**
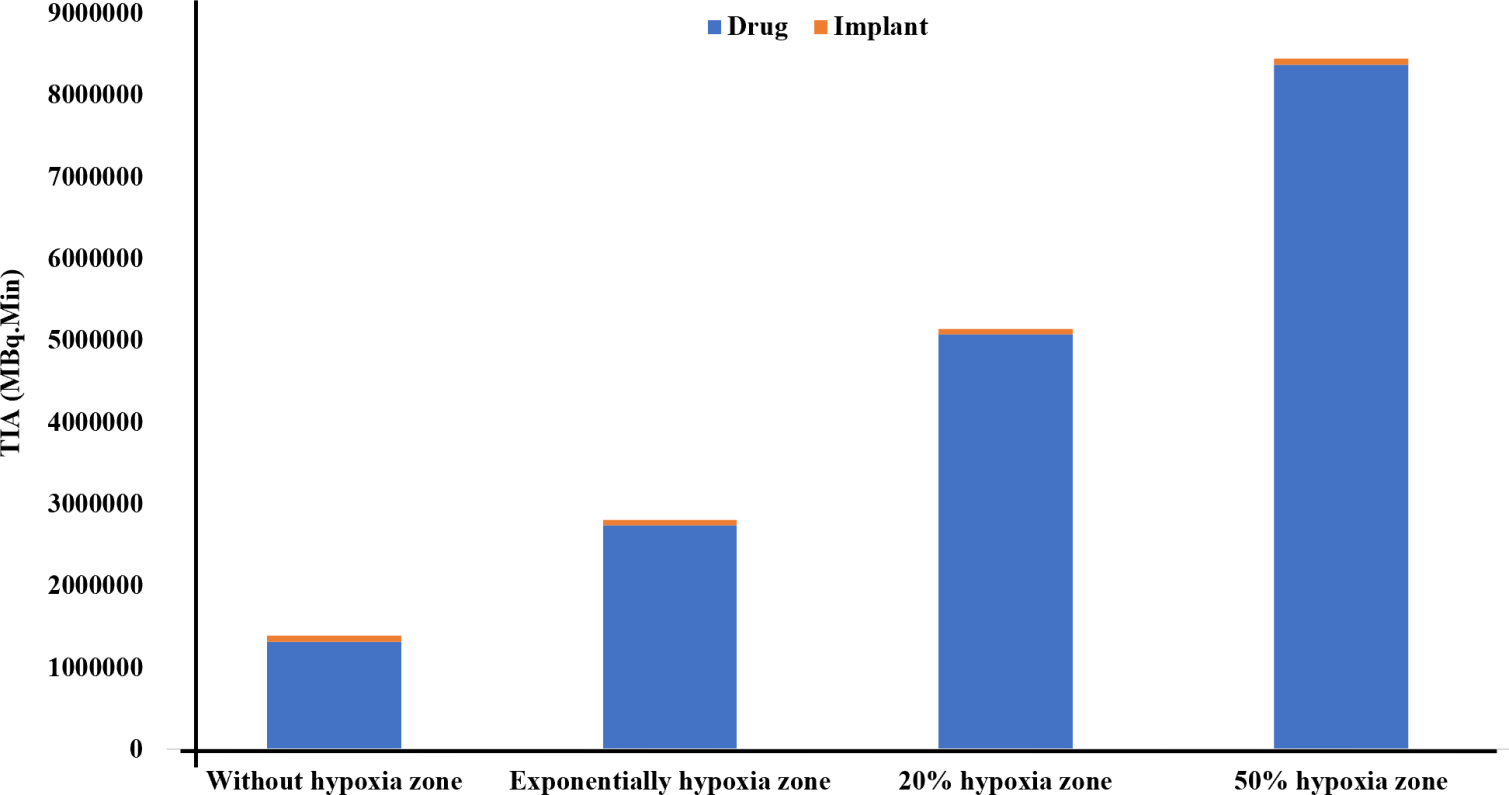
Comparison of the TIA [MBq.min] of the radiation source and the drug released from it considering the vascular density and extent of the hypoxia area. It is evident that the radiation source has a significantly lower TIA compared to the released drug. When the tumor experiences 50% hypoxia, TIA of the released drug increases sixfold compared to when there is no hypoxia present.

Finally, in this study, in the presence of 50% hypoxia in the tumor, the TIA increases six-fold compared to the absence of hypoxia (**Figure 4**). This significant rise in TIA indicates a substantial boost in the overall drug exposure within the tumor microenvironment over time. The heightened TIA highlights the importance of considering hypoxia as a key determinant affecting drug distribution and efficacy in the tumor. Such insights into the impact of hypoxia on drug delivery strategies can pave the way for more effective and precise treatment approaches for prostate cancer patients. When comparing the TIA of the radiation source (implant) and the released drug for the entire tumor, it becomes evident that the radiation source has a significantly inferior performance compared to the released drug. This is due to the fact that the radiation source remains fixed in place and does not reach a substantial portion of the tissue, unlike the released drug. Therefore, it can be anticipated that the radiation source alone will not generate an effective therapeutic response.

### 3.3 Effect of binding affinity

**Figure 5:**
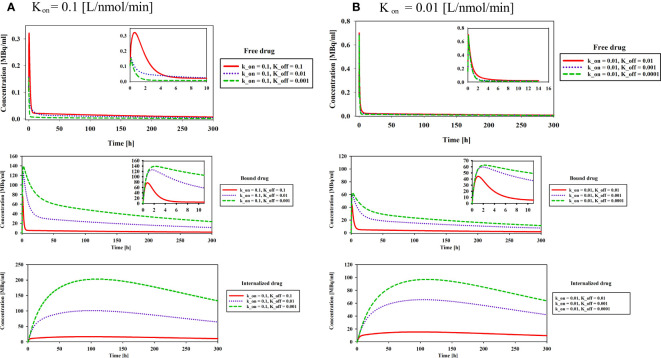
Effect of binding affinity on concentration of free, bound and internalized drug. Results reveal a noteworthy trend wherein the decrease in the dissociation constant K_D_, irrespective of the k_on_ values, is associated with higher peak concentrations of ^177^Lu-PSMA-617 bound to receptors and internalized within the cells.

**Figure 6:**
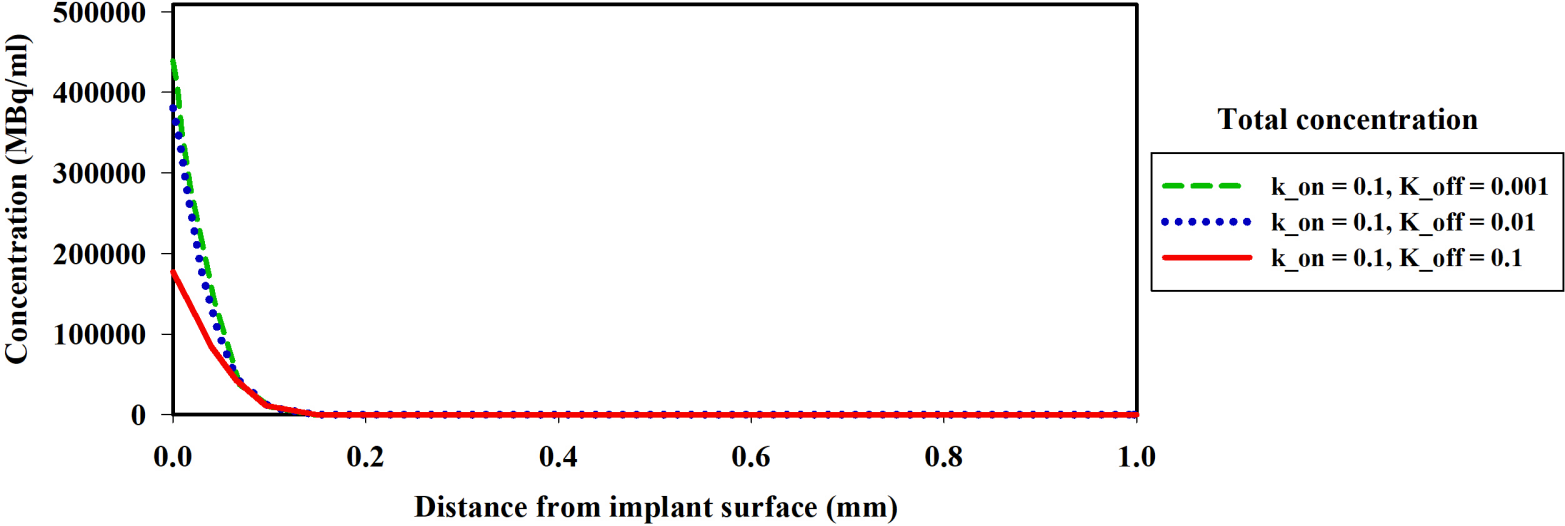
The spatial distribution of the concentration of the ^177^Lu-PSMA-617 released from the implant around the implant one hour after implantation in the tumor. A decrease in K_D_ leads to an increase in the total concentration of the drug. The k_off_ variations demonstrate that the highest total concentration is observed at k_off_=0.001 [1/min], indicating enhanced ligand penetration into tumor.

The association (k_on_) and dissociation (k_off_) rates play a significant role in drug-receptor interactions, impacting the concentration of ^177^Lu-PSMA-617 and behavior within the tumor microenvironment. The dissociation constant K_D_, defined as the ratio of the dissociation rate (k_off_) to the association rate (k_on_), characterizes the binding affinity (51, 52). The impact of K_D_ (1, 0.1 and 0.01[nM]), derived from various combinations of k_on_ and k_off_ as presented in **Table 1**, was examined to assess its influence on the time-integrated activity (TIA) of ^177^Lu-PSMA-617 in the tumor. To investigate the effect of K_D_ on TIA, a hypoxia-free condition is assumed with an initial ^177^Lu-PSMA-617 amount of 7.1×10^9^ [Bq] in the implant. The results of **Table 1** show that with the decrease of K_D_, the TIA has increased. This means that when the K_D_ decreases, the concentration of bound and internalized ^177^Lu-PSMA-617 in tumors increases over time (**Figure 5**). Based on **Figure 5**, a decrease in the dissociation constant K_D_ for both k_on_ values leads to higher peak concentrations of bound ^177^Lu-PSMA-617 to receptors and internalized drug. Moreover, a lower K_D_ implies slower clearance of the radiopharmaceutical agents from the receptors, indicating a stronger and more prolonged binding affinity of PSMA-617 to the receptors on the cell surface. Furthermore, **Figure 6** depicts the total concentration profile of ^177^Lu-PSMA concerning the distance from the implant surface at the one-hour mark following implantation in the tumor. According to **Figure 6**, when altering k_off_ from 0.1 to 0.001 [1/min] (while maintaining a constant association rate, kon, of 0.1 [L/nmol/min]), the peak total concentration is observed at k_off_=0.001 [1/min]. This implies that PSMA ligands have more time for entry into the cells under this dissociation rate. The present study’s findings align with those reported by Begum et al. (51), who utilized a physiologically based pharmacokinetic (PBPK) model to investigate the influence of K_D_ on the absorbed dose of ^177^Lu-PSMA-617. This shared observation suggests that the K_D_ parameter plays a pivotal role in determining the radiopharmaceutical’s concentration, which holds significant implications for the efficacy and therapeutic outcomes of PSMA-targeted therapies.

**Table 1:** Investigated Effect of different combinations of *k_on_* and *k_off_* on TIA.

KD(k_off_/k_on_)	K_on_	K_off_	TIA_Drug_ [MBq.min]	TIA_Implant_ [MBq.min]
**1**	0.01	0.01	12.4 × 10^5^	0.69 × 10^5^
**1**	0.1	0.1	13.5 × 10^5^	
**0.1**	0.1	0.01	81.04 × 10^5^	
**0.1**	0.01	0.001	52.2 × 10^5^	
**0.01**	0.1	0.001	160.7 × 10^5^	
**0.01**	0.01	0.0001	76.6 × 10^5^	

**Figure 7:**
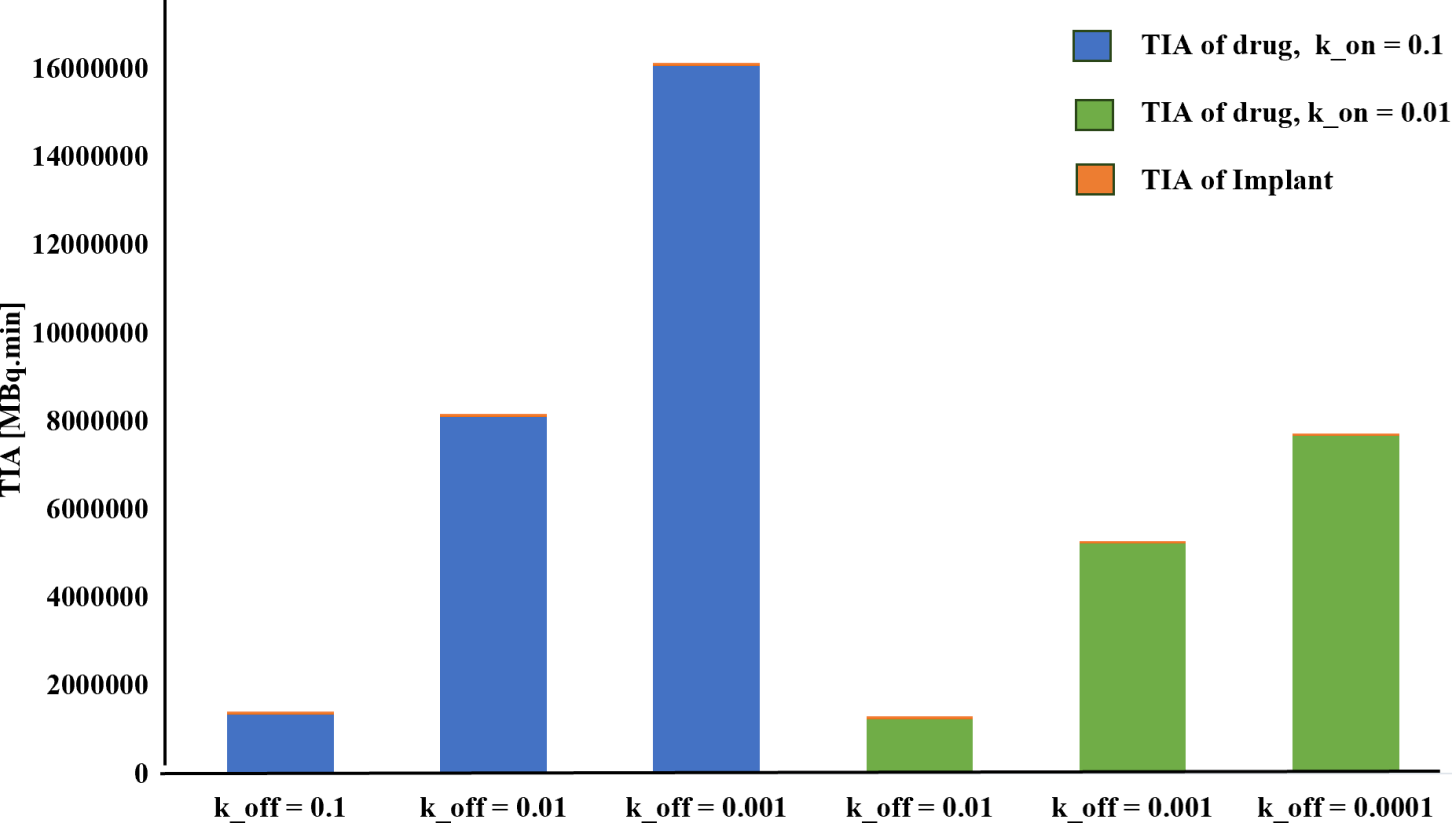
TIA comparison for different k_on. A higher time-integrated activity (TIA) is observed in the tumor for k_on_=0.1[L/nmol/min] compared to k_on_=0.01[L/nmol/min].

Furthermore, the results reveal that altering the association rate k_on_ exerts a more pronounced effect compared to adjustments in the dissociation rate k_off_. As depicted in **Figure 7** and **Table 1**, increasing the association rate k_on_ from 0.01 to 0.1 [L/nmol/min] resulted in a remarkable rise in the TIA from 12.4× 10^5^ [MBq.min] to 81.04× 10^5^ [MBq.min], with a fixed dissociation rate k_off_ of 0.01 [1/min]. Base on **Figure 7** and **Table 1**, 6-fold increase in TIA was observed when the dissociation rate k_off_ was changed from 0.01 to 0.0001 (with a fixed association rate k_on_ of 0.01 [L/nmol/min]) (from 12.4× 10^5^ to 76.6× 10^5^). It appears from the results that, by reducing the dissociation rate, the drug will have a greater chance of entering the cell, resulting in a higher amount of drug binding and entering the cell. In addition to the increase in association rate, the drug is able to bind to cell surface receptors faster, which results in less drug being removed from the tumor environment by the blood vessels. Furthermore, the comparison of the TIA of the radiation source also demonstrates that its value is significantly lower than the other results. Even if the drug exhibits different association and dissociation characteristics, leading to varying TIA values, it still outperforms the radiation source.

### 3.4 Effect of initial amount of ^177^Lu-PSMA

**Figure 8:**
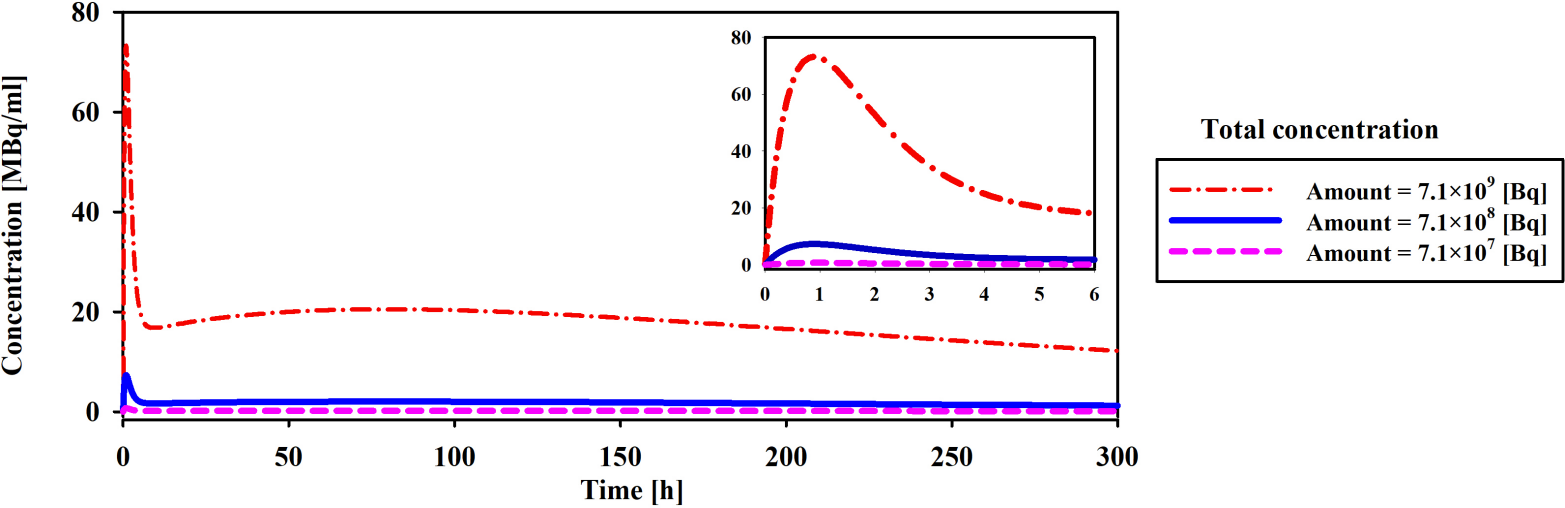
Comparison of total concentration of released drug from the implant for different initial amount of ^177^Lu-PSMA. The maximum total concentration in the tumor is achieved when the initial drug amount is set to 7.1×10^9^ [Bq].

**Figure 9:**
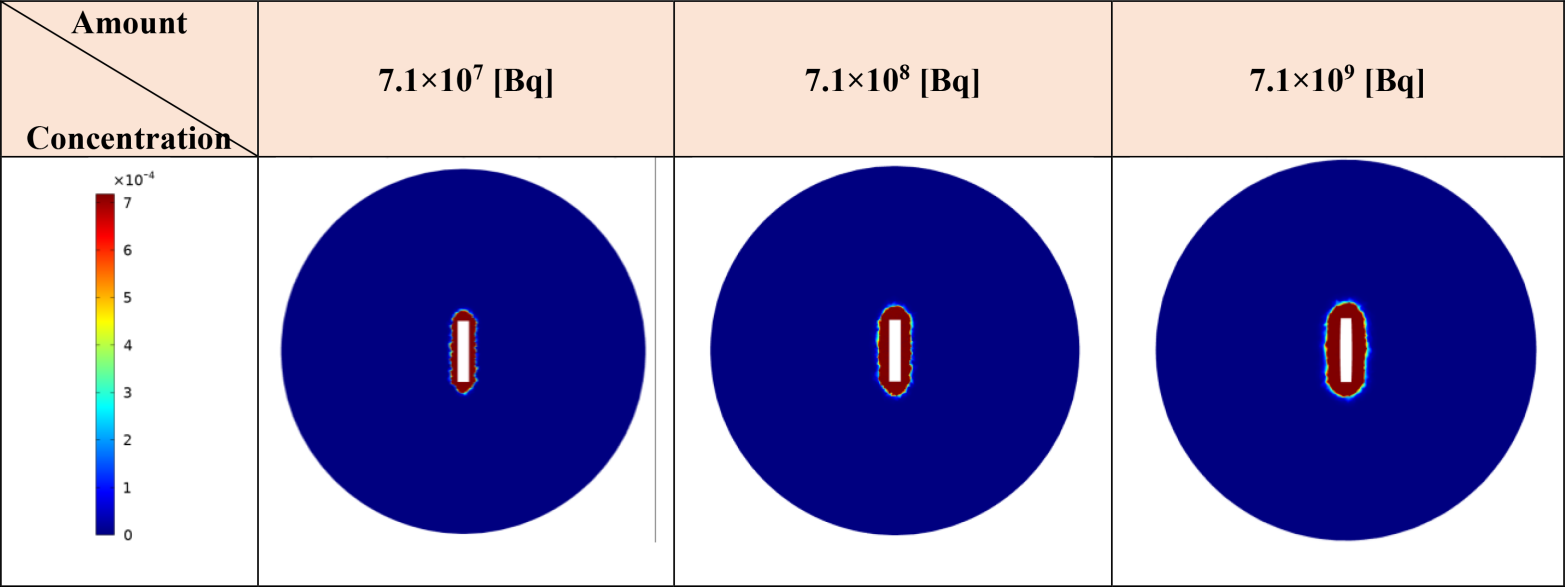
Modifications in the total concentration of released the radiopharmaceutical from implant [MBq/ml] are observed as ^177^Lu-PSMA quantity within the implant increases. Elevating the radiopharmaceutical amount results in a greater accumulation of the drug in the vicinity of the implant.

The initial drug amount loaded into the implant plays a pivotal role in determining treatment efficacy and potential damage to both tumor and surrounding tissues. In this study, varying the initial ^177^Lu-PSMA-617 amount 7.1×10^7^, 7.1×10^8^, and 7.1×10^9^ [Bq] led to distinct responses in terms of survival probability, concentration and damage to normal tissue. The study assumes a condition without hypoxia, with k_on_=0.046 [L/nmol/min] and k_off_=0.046 [1/min] (17). Based on **Figures 8** and **9**, it is evident that an initial ^177^Lu-PSMA-617 amount of 7.1×10^9^ [Bq] yields the highest total concentration within the tumor. It is important that the amount of radiopharmaceutical used results in maximal damage to the tumor tissue and minimum damage to the normal tissue. For the 7.1×10^7^ [Bq] initial radiopharmaceutical amount, the observed percentage of survival probability was 19%, indicating a moderate effect on tumor cell survival (**Table 2**). However, the impact on normal tissue was relatively low, with TIA calculated at 52×10^-4^ [MBq.min], suggesting minimal harm to healthy cells.

**Table 2:** Effect of amount of ^177^Lu-PSMA-617 on TIA.

Amount		7.1×10^7^ [Bq]	7.1×10^8^ [Bq]	7.1×10^9^ [Bq]
Time integrated activity (TIA) [MBq.min]	Released drug in tumor	13 × 10^3^	13 × 10^4^	13 × 10^5^
	Implant	0.68 × 10^3^	0.68 × 10^4^	0.68 × 10^5^
Absorbed dose [Gy]	Released drug in tumor	4.56	45.6	456
	Implant	0.23	2.3	23
Survival probability (%)	Released drug in tumor	19%	1.15 × 10^-45%^	0%
	Implant	96%	53%	3× 10^-14%^
Time integrated activity [MBq.min]	Drug in normal tissue	52 × 10^-4^	52 × 10^-3^	52 × 10^-2^

On the contrary, an initial radiopharmaceutical quantity of 7.1×10^8^ [Bq] led to an exceptionally low survival probability, approaching nearly zero percent, signifying a robust elimination of tumor cells (**Table 2**). Despite this potent effect on tumor tissue, normal tissues also experienced a higher TIA, with a calculated value of 52×10^-3^ [MBq.min]. This treatment not only exhibited excellent tumor-killing efficacy but also had a slightly increased impact on normal tissue. At the highest initial radiopharmaceutical amount of 7.1×10^9^ [Bq], the survival fraction dropped to 0%, illustrating complete eradication of tumor cells (**Table 2**). However, this extreme effectiveness was accompanied by a higher TIA in normal tissue, calculated at 52×10^-2^ [MBq.min], signifying more significant potential harm to healthy cells.

Overall, these findings underscore the delicate balance required in selecting the initial drug amount for radiopharmaceutical treatment. While higher initial drug amounts demonstrated superior tumor-killing potential, they also showed an elevated impact on normal tissues. The 7.1×10^8^ [Bq] initial drug amount exhibited promising outcomes, yielding a considerable TIA within the tumor and notably mitigating harm to normal tissue in contrast to 7.1×10^9^ [Bq].

When comparing the effects of the radiation source and the released drug on therapeutic response, considering the initial amount of the drug in the implant shown in **Table 2**, it becomes evident that the implant performs poorly as a radiation source in terms of treatment response. Increasing the initial amount of the drug loaded in the implant does improve the TIA and absorbed dose caused by the radiation source. However, these increases, particularly for the values of 7.1×10^7^ [Bq] and 7.1×10^8^ [Bq], do not result in significant cell death. For instance, with an initial amount of ^177^Lu-PSMA at 7.1×10^7^ [Bq], the released drug is capable of killing over 80% of cancer cells, while the implant, acting as the radiation source, only contributes 4% to cell death. In summary, these findings highlight the crucial role of drug release and its distribution within the tumor, emphasizing their significant contribution to therapeutic efficacy compared to using the implant solely as a radiation source.

### 3.5 Analyzing changes in the concentration of a drug

**Figure 10:**
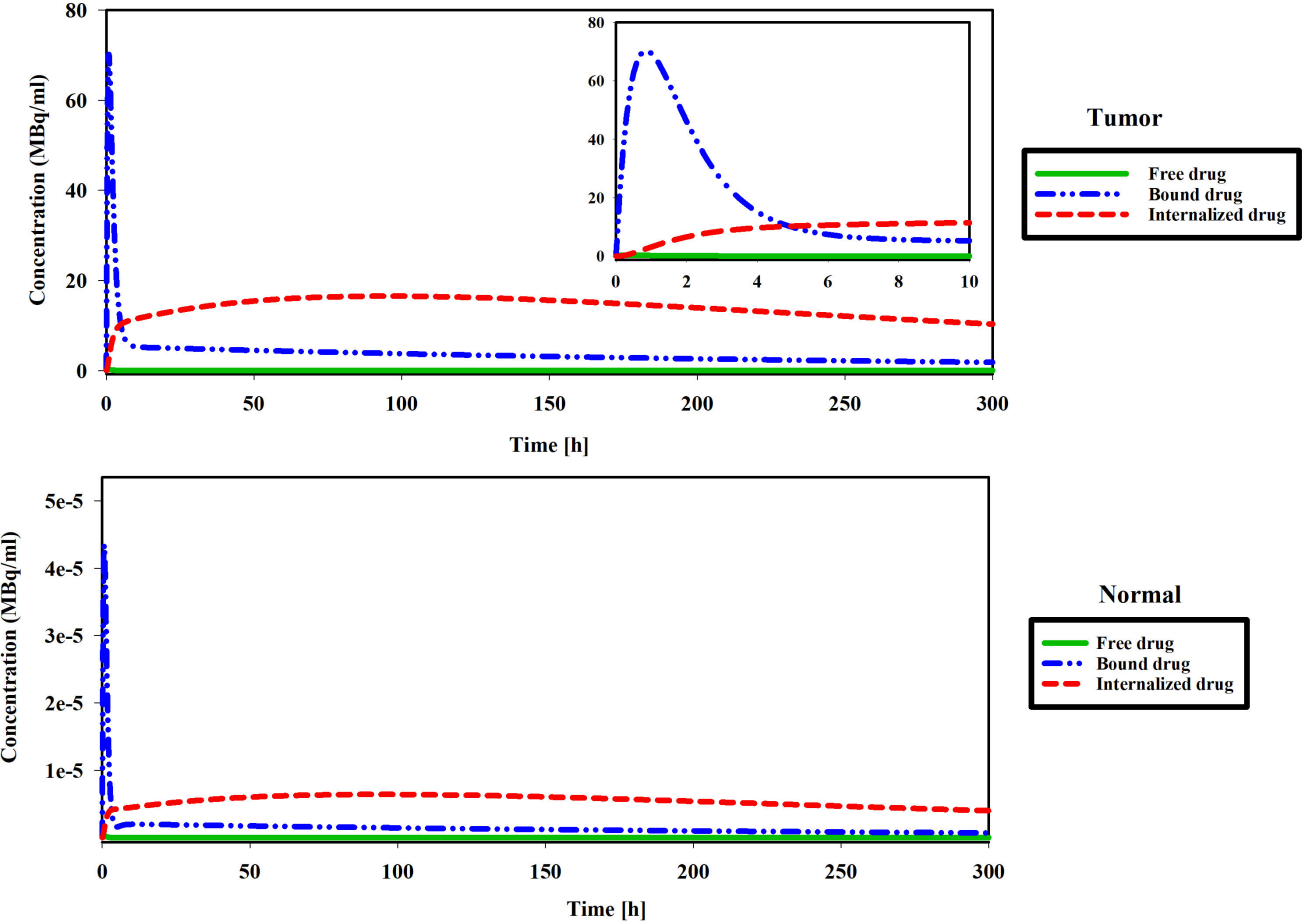
Figures show changes in the concentration of free, bound and internalized ^177^Lu-PSMA-617 at 300 h.

This phase of the investigation is dedicated to scrutinizing the spatial and temporal distribution patterns of radiopharmaceuticals within both the tumor and host tissue. The study operates under a hypoxia-free condition, characterized by k_on_=0.046 [L/nmol/min] and k_off_=0.046 [1/min] (17), with a loading of 7.1×10^9^ [Bq] of ^177^Lu-PSMA into the implant. **Figure 10** shows drug concentration in the different spaces. When the free radiopharmaceutical enters the interstitial space, it binds to receptors on the cell surface. Radiopharmaceutical initially bind to receptors rapidly. In addition, the lymphatic vessels of normal tissues remove some of the drugs from the interstitial space. Next, the PSMA ligand-protein complex is internalized into the tumor cells via endocytosis. Finally, drugs degrade or are released in cell. The concentrations of bound and free drugs reduce to an undetectable level. As can be seen in **Figure 10**, the drug concentrations in the tumor are several times higher than in normal tissue in all spaces (Free, Bound, and internalized drugs). Results show that the proposed treatment method has increased drug concentrations in tumors and reduced normal tissue damage. The absoreb dose in tumor was 456 [Gy] (SF in tumor: 0%).

**Figure 11:**
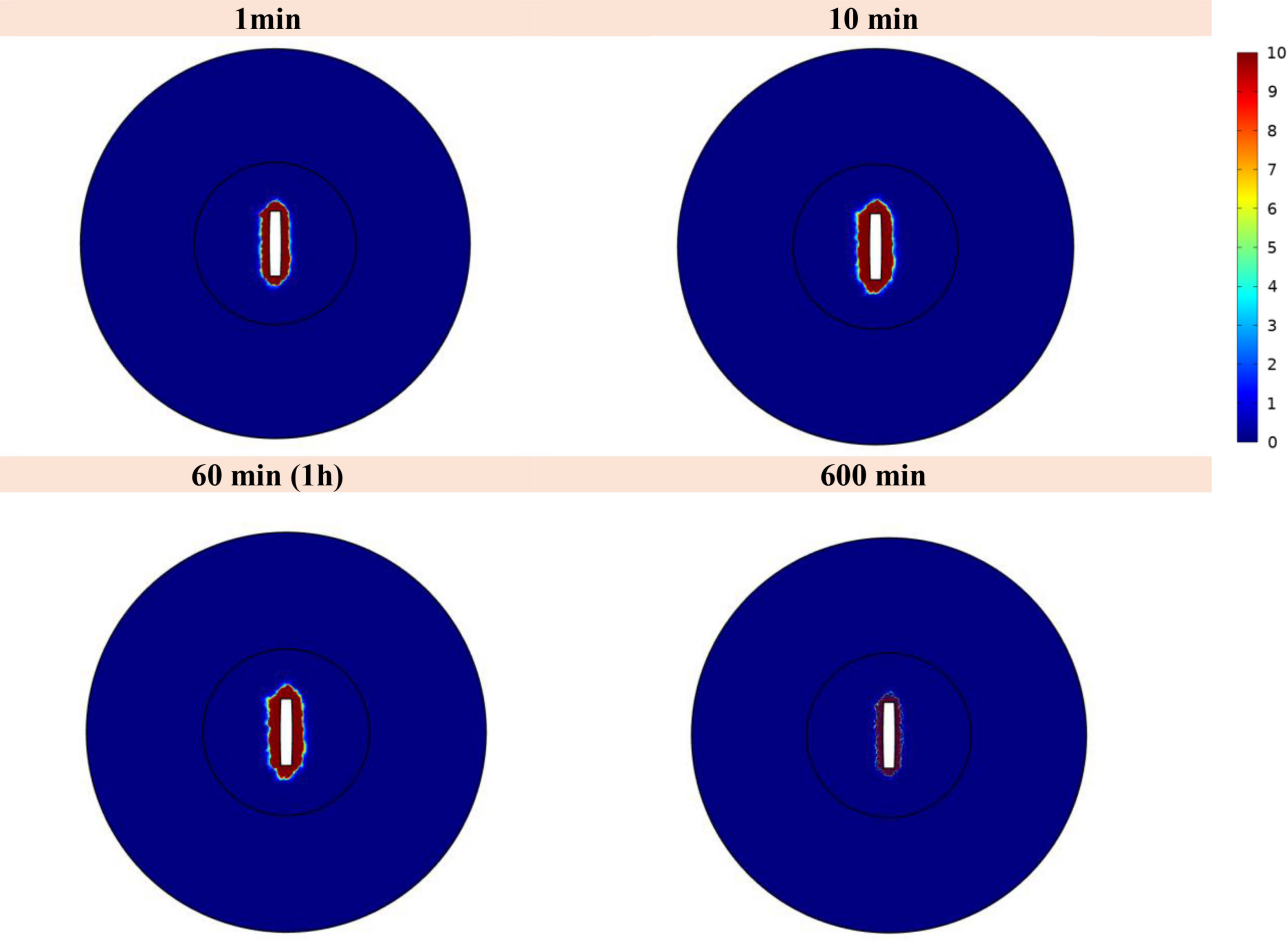
Standardized uptake value (SUV) index. The highest SUV is concentrated within the tumor area, displaying values several times greater than those detected in the adjacent normal tissue.

**Figure 11** illustrates the spatial distribution of ^177^Lu-PSMA within both the tumor and normal tissue at various temporal intervals. The figure distinctly portrays that the maximum Standardized Uptake Value (SUV) is localized within the tumor region, exhibiting values several times higher than those observed in the surrounding normal tissue. The constrained movement of high-molecular-weight PSMA ligands from the implant wall is a pivotal factor in this spatial distribution, contributing positively to minimizing tissue damage surrounding the tumor. It should be noted that due to the fact that there is no requirement for the radio-drug to enter the cell in order to damage its DNA, the treatment has been effective despite the fact that the drug has not been widely distributed in the tissue.

## 4 Discussion and conclusion

The use of implants for intratumoral drug delivery represents a promising approach in cancer treatment, offering several advantages over traditional intravenous injection methods (29). Implants allow for localized drug delivery, enabling targeted and sustained release of therapeutic agents directly into the tumor microenvironment (53). This localized drug delivery minimizes systemic exposure and reduces potential side effects, enhancing the overall safety profile of the treatment (54). Moreover, implants can overcome limitations associated with poor drug penetration and distribution within solid tumors, ensuring more efficient drug delivery to cancerous cells. This study presents a novel and comprehensive 3D computational model to simulate the dispersion and localized release of ^177^Lu-PSMA-617 after implanting a dual-release system within a solid tumor. The spatial analysis of the results demonstrates that the tumor region exhibits the maximum value of SUV, surpassing the SUV in the surrounding normal tissue by several-fold. The high molecular weight of peptides in PSMA ligands restricts their diffusion from the implant wall. Remarkably, this characteristic proves beneficial in limiting tissue damage around the tumor site. The observed 0% survival fraction for the tumor at an activity level of 7.40 MBq highlights the remarkable efficacy of this approach in eradicating tumor cells. Thus, the potential for accurate and successful tumor therapy is shown by this targeted radiopharmaceutical delivery strategy.

The present study seeks to find the effects of hypoxia region extension, binding affinity, and initial drug amount on ^177^Lu-PSMA-617 concentration in the tumor microenvironment. In general, the findings of this study demonstrate that elevated hypoxia levels are associated with a higher TIA within tumor due to microvascular density, resulting in enhanced drug availability for binding to PSMA receptors, reduced clearance of radiopharmaceutical agents from the interstitial space. The 50% hypoxia result in a six-fold increase in TIA within the tumor, relative to non-hypoxic conditions that ultimately promoting greater therapeutic effectiveness.

Another critical aspect under scrutiny is the binding affinity. The results indicate that reducing the dissociation constant (K_D_) while keeping k_on_ at 0.1 [L/nmol/min] and varying k_off_ from 0.01 to 0.0001[1/min] results in a substantial 6-fold increase in the TIA. The decrease in K_D_ and concurrent increase in bound concentration implies a heightened binding affinity of the radiopharmaceutical to the receptors present on the cell surface. Such an elevation in bound and internalized drug concentration results in reduced radiopharmaceutical clearance by vessels, potentially leading to decreased damage to other organs, such as the kidneys.

One of critical parameter under investigation is the initial radiopharmaceutical amount in the implant. This parameter directly influences the total drug concentration available for release and distribution within the tumor. The results allow researchers to determine the optimal amount of drug to be incorporated into the implant, ensuring maximal damage to the tumor while minimizing potential toxicity to surrounding healthy tissues. Among the considered initial radiopharmaceutical amounts of 7.1×10^7^, 7.1×10^8^, and 7.1×10^9^ [Bq], it becomes evident that the 7.1×10^8^ [Bq] amount demonstrates the most advantageous outcomes. Notably, the percentage of survival probability at 7.1×10^8^ [Bq] is significantly lower compared to that at 7.1×10^7^ [Bq], reaching a value of nearly 0%. Furthermore, despite its potent tumor cell elimination capabilities (reflected by the high survival probability), the TIA associated with the 7.1×10^8^ [Bq] amount is one order lower than that of 7.1×10^9^ [Bq], implying a more desirable balance in minimizing adverse effects to normal tissues. Consequently, the initial radiopharmaceutical amount of 7.1×10^8^ [Bq] stands out as a better choice for achieving therapeutic efficacy while mitigating potential harm to healthy tissues. In our studies, decay of labeled radiopharmaceuticals resulted in relatively negligible unlabeled pharmaceuticals (not shown) that would not pose competition for PSMA binding with radiopharmaceuticals. In addition, it is worth noting that in this study, free PSMA was not taken into consideration due to the local delivery approach. Conversely, when free PSMA is delivered at a high concentration in a small volume, it can lead to receptor saturation on the cell surface, resulting in a diminished therapeutic response. Therefore, by excluding free PSMA in this study, we can ensure that the receptors are not occupied and maximize the potential for an effective therapeutic outcome.

The results obtained from analyzing the TIA in different sections demonstrate that the distribution of radiopharmaceuticals has a more significant therapeutic effect compared to the implant, which also acts as a radiation source. This discrepancy can be attributed to the fact that the radiation source, despite having a high concentration, is only located in the center of the tumor. As a result, only a small portion of the tissue is exposed to radiation. On the other hand, the release of the radiopharmaceutical allows for its penetration into the tissue, resulting in a larger area of tissue being exposed to radiation. These findings highlight the superiority of this treatment approach over brachytherapy. Therefore, utilizing biodegradable implants containing radiopharmaceuticals can offer notable therapeutic performance in comparison to conventional radiotherapy methods.

The experimental design of this study, which revolves around a numerical simulation, is positioned as a pivotal guide for the formulation of optimal drug treatment protocols in future clinical trials. By delving into the controlled release and dispersion of ^177^Lu-PSMA through a dual-release implantable delivery system, nuanced insights are garnered into crucial parameters such as hypoxia extension, binding affinity, and initial drug amounts. Through this approach, a more comprehensive understanding of radiopharmaceutical therapy is facilitated, laying the groundwork for the development of meticulously tailored drug treatment strategies. The numerical nature of our study enables a detailed exploration of various dosage scenarios, providing a robust foundation for the optimization of treatment effectiveness in the intricate landscape of the tumor microenvironment. In this manner, the present numerical approach actively shapes the trajectory toward more effective and tailored drug treatments in the clinical trial setting.

In this study, one of limitations is the simplified tumor geometry, chosen based on literature for exploring drug and tumor-related parameters (29). Considering the depth of penetration and tissue distribution, a heterogeneous geometry minimally affects distribution, with only specific areas possibly having higher concentrations. Thus, the choice of simple geometry did not significantly influence the current results. Future studies will employ real tumor geometry for more comprehensive investigations.

Additionally, the utilization of implant-based drug delivery approaches comes with certain limitations. Intratumoral delivery of radiopharmaceuticals is confined to the local area, rendering it incapable of covering the entire body. Consequently, this therapeutic method is more suitable for treating pre-metastatic stages and localized tumors within the body. Moreover, tumors characterized by extensive angiogenesis present a challenge as the presence of blood vessels can swiftly remove the drug within the interstitial space, hindering effective drug delivery. To ensure optimal performance, it is essential to customize the design of the implant based on the specific characteristics of the tumor tissue. This involves optimizing the loaded capacity, number of inserted implants and release rate of the implant, considering the combined effects of burst and sustained release. By doing so, one can mitigate the risk of undesired outcomes such as elimination by blood/lymphatic microvessels. Overall, the design of the implant should align with the principles of personalized medicine to achieve favorable therapeutic outcomes.

## Data availability statement

The original contributions presented in the study are included in the article/**Supplementary Material**. Further inquiries can be directed to the corresponding author.

## Author contributions

AP: Conceptualization, Data curation, Formal Analysis, Investigation, Methodology, Resources, Software, Validation, Visualization, Writing – original draft. MSou: Conceptualization, Data curation, Formal Analysis, Investigation, Software, Validation, Writing – review & editing. AR: Data curation, Funding acquisition, Resources, Writing – review & editing. MSol: Project administration, Resources, Supervision, Writing – review & editing.
