# Supplementary material for: Localized radiotherapy of solid tumors using radiopharmaceutical loaded implantable system: insights from a mathematical model
Source: Front Oncol. 2024 Feb 26;14:1320371. doi: 10.3389/fonc.2024.1320371 (PMC10979490; doi:10.3389/fonc.2024.1320371)
Supplement: Supplementary file 1 [file DataSheet_1.pdf]

Supplementary file

Localized Radiotherapy of Solid Tumors Using Radiopharmaceutical Loaded Implantable System: Insights from a Mathematical Model

This document provides a detailed description of the mathematical model and parameters.

Methods

In this study, the following assumptions were considered: *i*) The implant is recognized as a radiation source, and its impact on the entire tumor is assessed by considering the rate of radiopharmaceutical release. Over time, as the drug is released, the effect of the radiation source diminishes. *ii*) Given the high density of cells in solid tumors, we considered a fixed number of available cell surface receptors. *iii*) The tumor has four microvessel-density zones: hypoxic, transitional hypoxic region, quiescent, and proliferative (figure 1S). Notably, hypoxic regions exhibit low microvascular density.

A

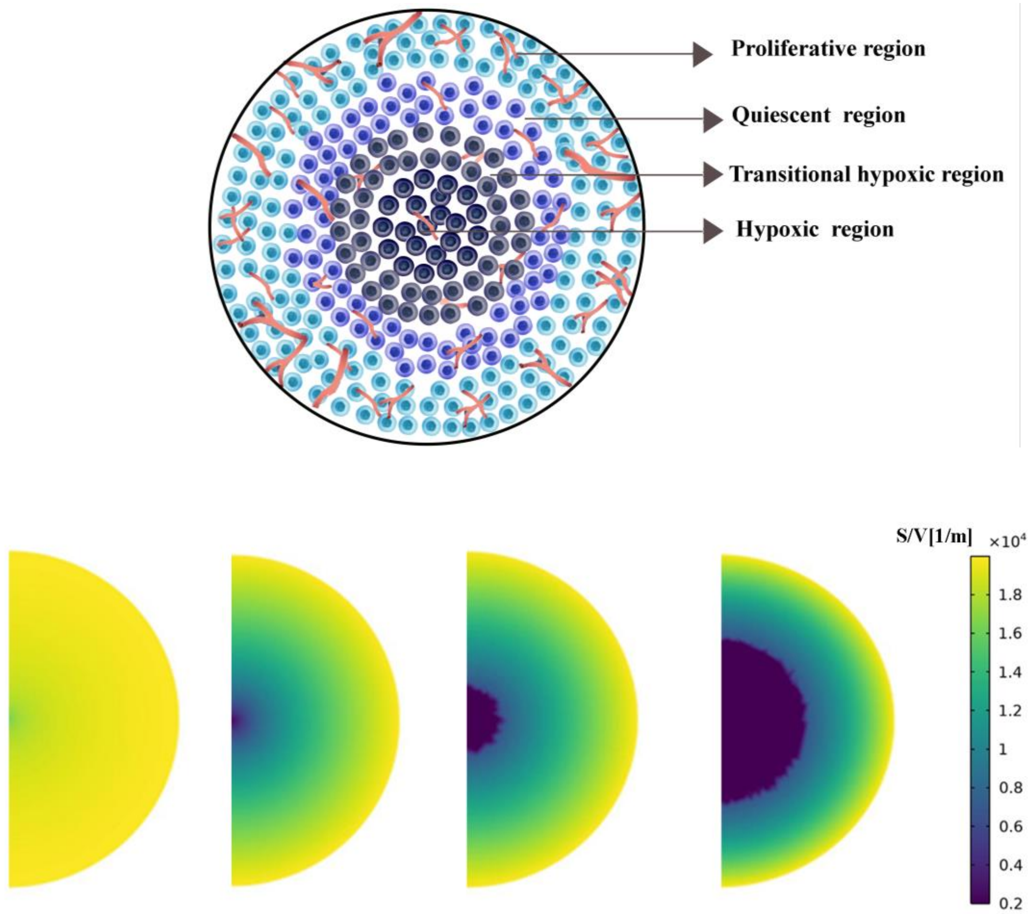

Figure S1. A) Comparison of tumor microenvironment regions based on vascular density: Proliferative region (high vascular density), Quiescent region (moderate vascular density), Transitional hypoxic regions (gradual decline in vascular density), and Hypoxic region (low vascular density). B) Four distinct scenarios for solid tumor is taken into account, each characterized by

varying degrees of hypoxia zones, including a case without any hypoxia region, an exponential hypoxia region, a 20% hypoxia zone and a 50% hypoxia zone.

1.1. Geometry and boundary conditions

The radius of the tumor and hosting tissue were set to 10 mm and 24 mm, respectively, in this investigation. The implant is situated at the center of the tumor and measures 0.75 mm in height and 8 mm in radius (Figure S2) (1) . Given the tumor size and the need to minimize harm to surrounding tissues, this specific implant size is identified as optimal. The modeling domain is simulated as a three-dimensional (3D) geometry. The complete mesh consists of 272316 domain elements, 12042 boundary elements, and 676 edge elements. Mesh independency has been attained through the examination of the total concentration of ^{177}Lu-PSMA. The boundary conditions for this investigation are summarized in Table 1.

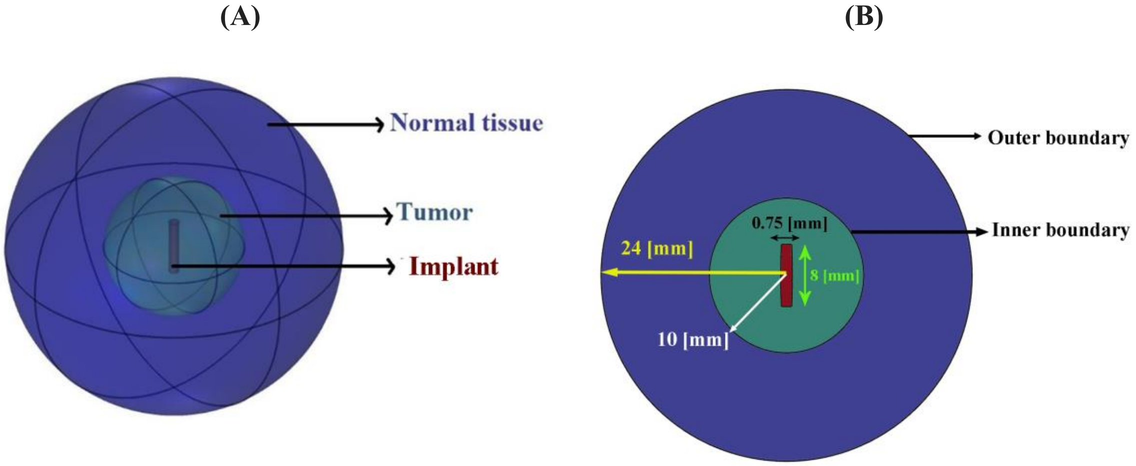

Figure S2. This schematic illustrates a cross-section of normal tissue, the implant, and the tumor. A) 3D profile of geometry, B) boundaries

Table 1. The boundary conditions of the present study for fluid flow and concentration distribution

Region	Fluid flow	Concentration
Boundary between tumor and normal tissues	$-K^t \nabla P_i\|_{\Omega^t} = -K^n \nabla P_i\|_{\Omega^n}$<br>$P_i\|_{\Omega^t} = P_i\|_{\Omega^n}$	$-D^t_{eff} \nabla C + v_i C\|_{\Omega^t} = -D^n_{eff} \nabla C + v_i C\|_{\Omega^n}$<br>$C\|_{\Omega^t} = C\|_{\Omega^n}$
Outer boundary	$P_i = 0$	$-n \cdot \nabla C = 0$

- Ω^t and Ω^n demonstrates the tumor and normal tissue at the boundary, respectively.
- n is the normal vector.

1.2. Governing equations

- **Implant**

In this model, a pre-made miniaturized implant is strategically positioned within a solid tumor by a catheter to facilitate the controlled release of ^{177}Lu-PSMA, allowing its diffusion into the surrounding tissue. Its structure and material type assume it to be a biodegradable implant. The present model considers dual-release implants. In the case of dual-release implants, release occurs in two distinctive phases: burst release and sustained release (1, 2). The burst release phase involves the rapid and immediate delivery of a significant amount of ^{177}Lu-PSMA, creating an instantaneous therapeutic impact. Subsequently, the sustained release phase ensues, characterized by a gradual and prolonged release of ^{177}Lu-PSMA over an extended period. The mechanism of release is based on diffusion, where drugs located near the implant surface are released explosively, and those in the center of the implant are released sustainably (3). The diffusion process commences once interstitial fluid permeates the implant. In the present study, to prevent high local concentration and entry into the bloodstream, the contribution of burst release is recognized, and the fraction of drug released during the explosion phase is set at 0.1(1).

This approach ensures a controlled and targeted release of the drug, optimizing therapeutic effectiveness while mitigating potential side effects (1, 4). However, the intricate challenges inherent in designing and optimizing implants for specific tumors must be acknowledged. For instance, an inaccurate determination of the burst release rate may lead to toxicity or excessive drug clearance from the tumor. In such circumstances, computational pharmacodynamic and pharmacokinetic modeling emerges as an indispensable tool.

The flux, which is the amount of ^{177}Lu-PSMA released across the implant surface over time is calculated as follows (1):

$$R_{177lu-PSMA}(t) = \frac{M_0.w_\infty}{A}(f.k_f e^{-k_f t} + (1-f).k_s e^{-k_s t}) \quad \text{(S1)}$$

where M_0 represents the total quantity of filled drug in the device, W represents the total fraction of drug released at steady state, and f represents the fraction of drug released during the explosion phase. The parameters k_f and k_s represent burst and sustained release rate constants, respectively. The total quantity of ^{177}Lu-PSMA in the implant was initially set at 7.1×10^9 [Bq], and it decreases over time based on the release rate. Figure S3 shows drug release profile from the implant.

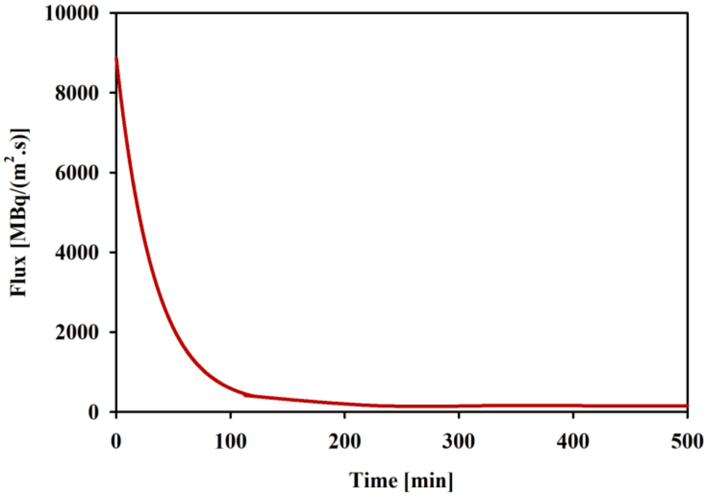

Figure S3. Drug release profile from implant. The release profile is characterized by two distinct phases: an initial burst release, marked by rapid and immediate drug delivery, followed by a sustained release phase, indicating a gradual and prolonged release of the therapeutic agent.

- **Interstitial fluid transport**

The movement of therapeutic agents within biological tissue via convection relies on the fluid flow equation within tissues. Given that biological tissues exhibit a porous nature, darcy's equation are employed to characterize the flow of fluids within them. Darcy's equation is used to determine the connection between interstitial fluid pressure (IFP) and interstitial fluid velocity (IFV). Consequently, the following definition applies to the fluid equation in a tissue (5, 6):

$$\nabla P_i = -\left(\frac{\mu}{k}\right) v_i \tag{S2}$$

$$v_i = -\kappa \nabla P_i \tag{S3}$$

where v and p_i are the IFV and IFP, respectively. κ is the hydraulic conductivity of the tissue and μ is viscosity. In porous media like tissue, a capillary network is the source (φ_B) and a lymphatic drainage system is the sink (φ_L). The continuity equation is calculated as follows.

$$\nabla \cdot v_i = \varphi_B - \varphi_L \tag{S4}$$

In accordance with Starling's law, φ_B and φ_L can be determined:

$$\varphi_B = L_P \left(\frac{S}{V}\right)\left(P_B - P_i - \sigma_s\left(\pi_B - \pi_i\right)\right) \tag{S5}$$

$$\varphi_L = L_{PL} \left(\frac{S}{V}\right)_L \left(P_i - P_L\right) \tag{S6}$$

where L_P is the hydraulic conductivity of the microvessel wall, P_B is represent the vascular pressures, $\frac{S}{V}$ is the surface area of blood vessels per unit volume of tissue. σ_S is the average osmotic reflection coefficient, π_B is the osmotic pressure of the plasma, and π_i is that of interstitial fluid. L_{PL} is the hydraulic conductivity of the lymphatic wall. The $\frac{S_L}{V}$ and $P_L$are represent the surface area of lymphatic vessels per unit volume of tissue, and the intra-lymphatic pressure. Finally, the following equations can be used to calculate interstitial fluid transport:

$$-\kappa\nabla^2 P_i = \begin{cases} \frac{L_P S}{V}\left(P_B - P_i - \sigma_s\left(\pi_b - \pi_i\right)\right) - \frac{L_{PL} S_L}{V}\left(P_i - P_L\right) & \text{Normal tissue} \quad \text{(S7)} \\ \frac{L_P S}{V}\left(P_B - P_i - \sigma_s\left(\pi_b - \pi_i\right)\right) & \text{Tumor tissue} \quad \text{(S8)} \end{cases}$$

- **[177]Lu-PSMA concentration**

The computational domain of the model includes both malignant and healthy tissues. A drug that inhibits cancer is released from the implant and diffuses into the surrounding tissue based on convection and diffusion mechanisms. There are several pharmacokinetic processes that affect therapeutic agents' distribution, including absorption into cells, receptor binding and unbinding, biodegradation, and physical decay. Convection-Diffusion-Reaction (CDR) equations describe radiopharmaceutical distribution. In extracellular space, the mass transport of free radiopharmaceuticals can be summarized by the equation below ($C_{labeled\_F}$):

Labeled (i.e. radiolabeled; hot):

$$\frac{\partial C_{labeled\_F}}{\partial t} = -\overbrace{v\nabla C_{labeled\_F}}^{convection} + \overbrace{D_{eff}\nabla^2 C_{labeled\_F}}^{diffusion} - \frac{(R_{max})k_{on}C_{labeled\_F}}{\psi} + k_{off}C_B - FC_{labeled} - \lambda_{lu177}C_{labeled\_F} \quad \text{(S9)}$$

Where the loss rate of free-drug (*F*) due to drainage in the lymphatic vessels and elimination by the blood capillaries.

$$-k_i\nabla^2 P_i = \begin{cases} F_{Fi} & \text{tumour} \\ F_{Fi} - \varphi_L, & \text{Normal tissue} \end{cases}$$

The loss rate of free-drug due to elimination by blood vessels (F_{Fi}) can be expressed as follows:

$$F_{Fi} = \frac{P_{Fi} S_{vi}}{V_i} \quad \text{(S10)}$$

where P_{Fi} is the permeability coefficient of the blood vessel wall to free-drug in (m/s). Furthermore the physical decay of radionuclide is λ_{lu177}. The association and dissociation rates are k_{on} and $k_{off.}$ and, R_{max} is maximum of receptor density on cell surface.

- **Bound drug (C_B) concentration:**

The Bound concentration is the amount of ^{177}Lu-PSMA-617 that is bound to cell receptors on the surface of cancerous cells. This parameter is essential in targeted radionuclide therapy for prostate cancer, as it indicates the extent of effective targeting and binding of ^{177}Lu-PSMA-617 to PSMA-expressing cancer cells. Equation 4 is utilized to calculate the bound drug concentration (7, 8):

Labeled: (S11)

$$\frac{\partial C_B}{\partial t} = \frac{(R_{max})k_{on}C_{labeled-F}}{\psi} - k_{off}C_B - k_{\text{int}}C_B - \lambda_{\text{lu177}}C_{\text{B}}$$

Ψ refers to the amount of tumor that a drug can reach. k_{int} is the internalization rate. K_{on} and k_{off} influence binding and unbinding speed.

- **Intracellular concentration:**

Upon ligand-receptor binding, the ensuing complex is internalized by the cancer cells through endocytosis, thereby facilitating its accumulation within the intracellular milieu(9). The intracellular drug concentration (C_{int}) follows the principle of mass conservation, as depicted in the equation below (7, 8):

Labeled: (S12)

$$\frac{\partial C_{\text{int}}}{\partial t} = k_{\text{int}}C_B - k_{\text{deg}}C_{\text{int}} - \lambda_{\text{lu177}}C_{\text{int}}$$

where K_{deg} is the release/ degradation rate (8, 10, 11).

- **Absorbed Dose, Cell Survival Probability and SUV (8)**

In radiopharmaceutical therapy, the absorbed dose is the amount of ionizing radiation energy deposited per unit mass of a material (12). It is a crucial parameter used to quantify the energy absorbed by tissues or organs in the body when exposed to radiation(12). To calculate the absorbed for tumor the following equations are used (10, 11, 13):

$$\dot{D}_i(t) = A_i(t) \cdot S_{i \leftarrow i} = A_0 \cdot a_i(t) \cdot S_{i \leftarrow i} \quad \text{(S13)}$$

$$D_i(T) = \int_0^T \dot{D}_i(t)dt = A_0 \cdot \tilde{a}_i(T) \cdot S_{i \leftarrow i} \quad \text{(S14)}$$

where $D_i(T)$ is the cumulated absorbed dose in a target region at T. T is the radiation exposure time, A_0 is the total activity, $\tilde{a}_i(T)$ is the time-integrated activity coefficient for a source region at time and $S_{i \leftarrow i}$ is the dose factor from a source region.

Cell Survival Probability is the probability that a given cell will survive after being exposed to a specific dose of ionizing radiation (14). Linear quadratic models are used to calculate the probability that a cell will survive (8):

$$P_S = e^{-\alpha D - \beta D^2} \tag{S15}$$

In this equation, D [Gy] denotes the absorbed dose, while α and β are coefficients known as the linear and quadratic parameters. These parameters are essential in characterizing the biological response of cells to ionizing radiation. The linear term (αD) captures the response proportional to the dose, while the quadratic term (βD^2) accounts for the dose-squared dependence (8). Understanding these parameters provides valuable insights into the complex interplay between radiation dose and cellular survival, allowing for a more nuanced evaluation of the impact of ionizing rays on biological systems.

The standardized uptake value (SUV) index is used to quantify radiopharmaceutical absorption. SUV is calculated as the body weight-normalized ratio of the total tissue radioactivity concentration examined in a location of interest to the radioactivity injected into the body as follows (15):

$$SUV = \frac{concentration\ of\ 177\ Lu\_PSMA}{injected\ radioactivity} \times Body\ Weight \tag{S16}$$

2.3. Numerical approach, and parameters

Tables 2 and 3 show the value of the parameters found in the current study. To run the simulation, COMSOL Multiphysics 5.6 (COMSOL Inc., Burlington, MA, United States) was used. For the solution of this issue, there are two distinct procedures that can be followed: steady-state and time-dependent. During the steady-state step, Darcy's law is applied in order to get the IFP and IFV values. The next step in the procedure is to solve equations describing the time-dependent distribution of drugs. With a time, step of 0.1 h and a relative tolerance of 0.001, a partitioning method has been employed to solve the equations.

The present study examines the effect of the following parameters on the time-integrated activity (TIA) and absorbed dose in the tumor:

- Effect of binding affinity
- Effect of initial amount of ^{177}Lu-PSMA
- Effect of extension of hypoxia region

The function of vessels as sinks is predicted by the present mathematical models, thus it is crucial to look at this parameter and how it affects treatment. In this study, various hypoxic regions within solid tumors are investigated. These include areas without hypoxia, regions with 20% hypoxia, zones with 50%

hypoxia, and exponentially hypoxic regions, each providing insights into the microvascular density within tumors.

**Table 2.** Values of the parameters used in the model for ^{177}Lu-PSMA and implant.			
**Parameter**	**Value**	**Ref**	**Definition**
D_{eff}	8.7×10^{-7} [cm^2 s^{-1}]	(8)	Coefficient of effective diffusion
R_{max}	50 [$nmol\cdot l^{-1}$]	(11)	receptors density
ψ	0.4	(8)	Fractional interstitial volume
λ_{lu177}	7.15×10^{-5} [Min^{-1}]	(11)	Physical decay ^{177}Lu
K_{int}	0.001[Min^{-1}]	(11)	Internalization rate of the cell through the receptors
k_{on}	0.046 [L/nmol/min]	(11)	Drug binding rate to cell receptors
k_{off}	0.046[Min^{-1}]	(11)	Drug unbinding rate from cell receptors
k_{rel}	2×10^{-4} [Min^{-1}]	(11)	Release rate
P	3.3×10^{-4} [cm s^{-1}]	(8)	Vessel wall permeability
R_f	1	(8)	Molecule/Carrier movement coefficient
f	0.1	(1)	The fraction of drug released during the explosion phase
k_s	1×10^{-6} [s^{-1}]	(1)	Sustained release rate
kf	5×10^{-4} [s^{-1}]	(1)	Burst release rate
w_∞	1	(1)	The total fraction of drug released at steady state

**Table 3.** Values of the parameters used in the modeling of interstitial fluid flow.			
**Parameter**	**Tissue**	**Value**	**Reference**
K [cm^2/mmHgs]	Normal	8.53×10^{-9}	(6, 16)
	Tumor	4.13×10^{-8}	(6, 16)
Lp [cm/mmHgs]	Normal	0.36×10^{-7}	(6, 17)
	Tumor	2.80×10^{-7}	(6, 17)
L_{PL} S_L/V [1/mmHg s]	Normal	1.33×10^{-5}	(17, 18)
P_B[mmHg]	Both	15.6	(6)
P_l	normal	0	(17, 18)
S/V[cm^{-1}]	Normal	70	(6, 19)
	Tumor	200	(6, 19)
Π_B[mmHg]	Both	20	(6)

Table 3. Values of the parameters used in the modeling of interstitial fluid flow.			
**Parameter**	**Tissue**	**Value**	**Reference**
Π_i[mmHg]	Normal	10	(6)
	Tumor	15	(6)
σ_s	Normal	0.91	(6)

References

1. Al-Zu'bi M, Mohan A. Modelling of combination therapy using implantable anticancer drug delivery with thermal ablation in solid tumor. Scientific reports. 2020;10(1):19366.
2. Musmade N, Jadhav A, Moin P, Patil S, Gupta A. An overview of in situ gel forming implants: current approach towards alternative drug delivery system. J Biol Chem Chron. 2019;5:14-21.
3. Stewart SA, Domínguez-Robles J, Donnelly RF, Larrañeta E. Implantable polymeric drug delivery devices: classification, manufacture, materials, and clinical applications. Polymers. 2018;10(12):1379.
4. Jeganathan S, Budziszewski E, Hernandez C, Dhingra A, Exner AA. Improving treatment efficacy of in situ forming implants via concurrent delivery of chemotherapeutic and chemosensitizer. Scientific Reports. 2020;10(1):6587.
5. Moradi Kashkooli F, Souri M, Tavakkoli J, C. Kolios M. A spatiotemporal computational model of focused ultrasound heat-induced nano-sized drug delivery system in solid tumors. Drug Delivery. 2023;30(1):2219871.
6. Soltani M, Chen P. Numerical modeling of fluid flow in solid tumors. PloS one. 2011;6(6):e20344.
7. Begum NJ, Glatting G, Wester H-J, Eiber M, Beer AJ, Kletting P. The effect of ligand amount, affinity and internalization on PSMA-targeted imaging and therapy: A simulation study using a PBPK model. Scientific reports. 2019;9(1):1-8.
8. Birindelli G, Drobnjakovic M, Morath V, Steiger K, D'Alessandria C, Gourni E, et al. Is Hypoxia a Factor Influencing PSMA-Directed Radioligand Therapy?—An In Silico Study on the Role of Chronic Hypoxia in Prostate Cancer. Cancers. 2021;13(14):3429.
9. Sgouros G, Bodei L, McDevitt MR, Nedrow JR. Radiopharmaceutical therapy in cancer: clinical advances and challenges. Nature reviews Drug discovery. 2020;19(9):589-608.
10. Begum NJ, Thieme A, Eberhardt N, Tauber R, D'Alessandria C, Beer AJ, et al. The effect of total tumor volume on the biologically effective dose to tumor and kidneys for 177Lu-labeled PSMA peptides. Journal of Nuclear Medicine. 2018;59(6):929-33.
11. Kletting P, Schuchardt C, Kulkarni HR, Shahinfar M, Singh A, Glatting G, et al. Investigating the effect of ligand amount and injected therapeutic activity: a simulation study for 177Lu-labeled PSMA-targeting peptides. PLoS One. 2016;11(9):e0162303.
12. O'Donoghue J, Zanzonico P, Humm J, Kesner A. Dosimetry in radiopharmaceutical therapy. Journal of Nuclear Medicine. 2022;63(10):1467-74.
13. Hindorf C. Internal Dosimetry. Chapter 18. 2014.
14. Spoormans K, Crabbé M, Struelens L, De Saint-Hubert M, Koole M. A Review on Tumor Control Probability (TCP) and Preclinical Dosimetry in Targeted Radionuclide Therapy (TRT). Pharmaceutics. 2022;14(10):2007.
15. Kashkooli FM, Abazari MA, Soltani M, Ghazani MA, Rahmim A. A spatiotemporal multi-scale computational model for FDG PET imaging at different stages of tumor growth and angiogenesis. Scientific reports. 2022;12(1):10062.

16. Soltani M, Souri M, Moradi Kashkooli F. Effects of hypoxia and nanocarrier size on pH-responsive nano-delivery system to solid tumors. Scientific Reports. 2021;11(1):19350.
17. Pishko GL, Astary GW, Mareci TH, Sarntinoranont M. Sensitivity analysis of an image-based solid tumor computational model with heterogeneous vasculature and porosity. Annals of biomedical engineering. 2011;39(9):2360-73.
18. Shahvandi MK, Souri M, Tavasoli S, Kashkooli FM, Kar S, Soltani M. A comparative study between conventional chemotherapy and photothermal activated nano-sized targeted drug delivery to solid tumor. Computers in Biology and Medicine. 2023;166:107574.
19. Souri M, Soltani M, Moradi Kashkooli F. Computational modeling of thermal combination therapies by magneto-ultrasonic heating to enhance drug delivery to solid tumors. Scientific reports. 2021;11(1):19539.
